# Potent inhibition of tumour cell proliferation and immunoregulatory function by mitochondria-targeted atovaquone

**DOI:** 10.1038/s41598-020-74808-0

**Published:** 2020-10-21

**Authors:** Gang Cheng, Micael Hardy, Paytsar Topchyan, Ryan Zander, Peter Volberding, Weiguo Cui, Balaraman Kalyanaraman

**Affiliations:** 1 Department of Biophysics, Medical College of Wisconsin, 8701 Watertown Plank Road, Milwaukee, WI 53226 USA; 2 Free Radical Research Center, Medical College of Wisconsin, 8701 Watertown Plank Road, Milwaukee, WI 53226 USA; 3 Microbiology and Immunology, Medical College of Wisconsin, 8701 Watertown Plank Road, Milwaukee, WI 53226 USA; 4 Cancer Center, Medical College of Wisconsin, 8701 Watertown Plank Road, Milwaukee, WI 53226 USA; 5 Center for Disease Prevention Research, Medical College of Wisconsin, 8701 Watertown Plank Road, Milwaukee, WI 53226 USA; 6 Aix Marseille Univ, CNRS, ICR, UMR 7273, 13013 Marseille, France; 7 Versiti Blood Research Institute, 8733 Watertown Plank Road, Milwaukee, WI 53226 USA

**Keywords:** Breast cancer, Lung cancer, Pancreatic cancer, NMR spectroscopy, Biological techniques, Biophysics, Drug discovery, Molecular biology, Molecular medicine

## Abstract

The FDA-approved prophylactic antimalarial drug atovaquone (ATO) recently was repurposed as an antitumor drug. Studies show that ATO exerts a profound antiproliferative effect in several cancer cells, including breast, ovarian, and glioma. Analogous to the mechanism of action proposed in parasites, ATO inhibits mitochondrial complex III and cell respiration. To enhance the chemotherapeutic efficacy and oxidative phosphorylation inhibition, we developed a mitochondria-targeted triphenylphosphonium-conjugated ATO with varying alkyl side chains (Mito_4_-ATO, Mito_10_-ATO, Mito_12_-ATO, and Mito_16_-ATO). Results show, for the first time, that triphenylphosphonium-conjugated ATO potently enhanced the antiproliferative effect of ATO in cancer cells and, depending upon the alkyl chain length, the molecular target of inhibition changes from mitochondrial complex III to complex I. Mito_4_-ATO and Mito_10_-ATO inhibit both pyruvate/malate-dependent complex I and duroquinol-dependent complex III-induced oxygen consumption whereas Mito_12_-ATO and Mito_16_-ATO inhibit only complex I-induced oxygen consumption. Mitochondrial target shifting may have immunoregulatory implications.

## Introduction

**Figure 1:**
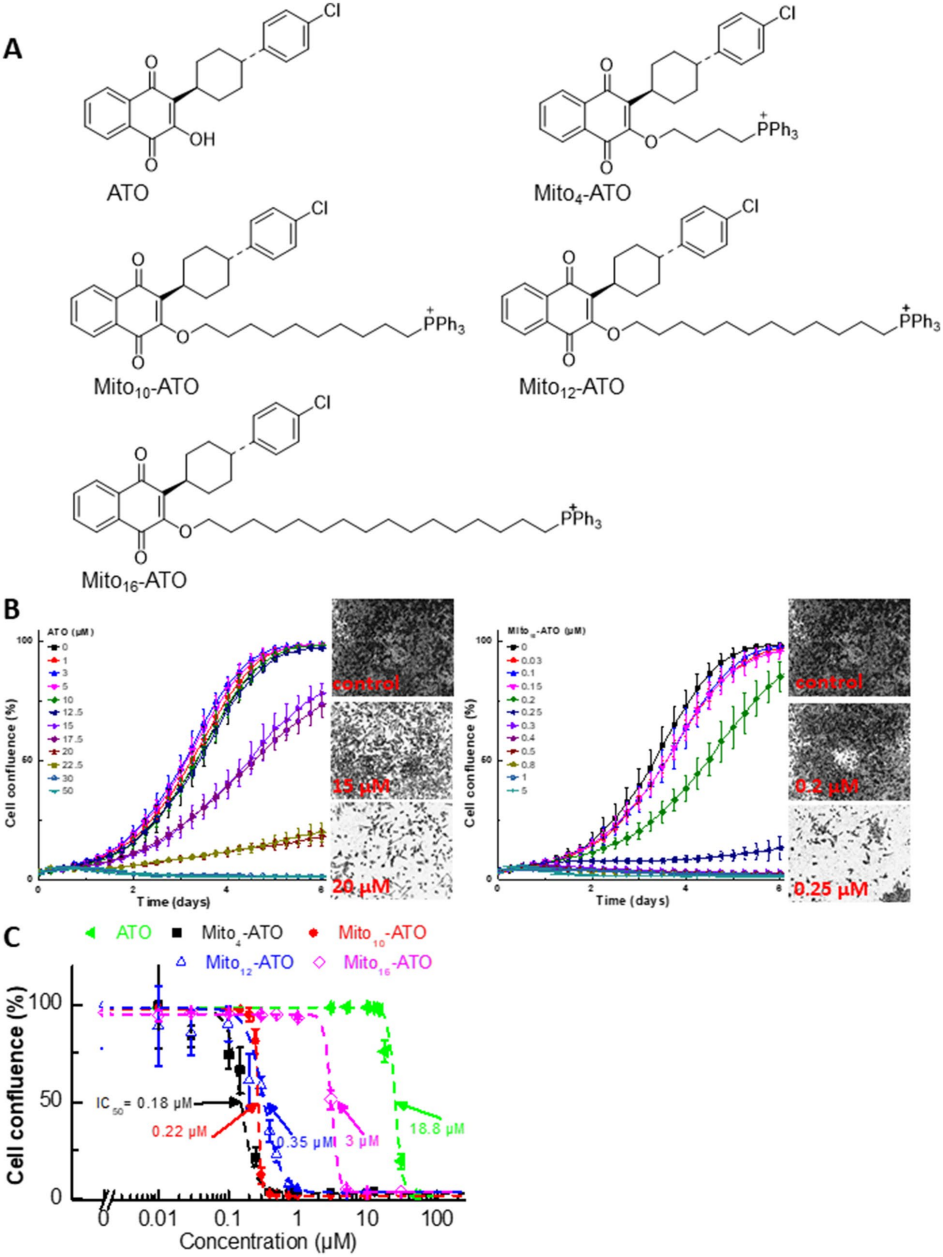
Effects of ATO, Mito-ATO analogs, and related analogs on proliferation of MiaPaCa-2 cells. (**A**) Chemical structures of ATO and Mito-ATO analogs. (**B**) Effect of ATO on the proliferation of human pancreatic cancer cells was compared with that of Mito_10_-ATO in the IncuCyte Live-Cell Imager. MiaPaCa-2 cells were treated with ATO and Mito_10_-ATO. Cell proliferation was monitored in real-time with the continuous presence of indicated treatments until the end of each experiment. Created using NIH public domain image processing program, ImageJ^38^. (**C**) Cell confluence (as control groups reach 98% confluency) is plotted against concentrations of ATO and Mito-ATO analogs. Dashed lines represent the fitting curves used to determine their IC_50_ values as indicated.

Atovaquone (ATO), a hydroxy-1,4-naphthoquinone analog of ubiquinone (Q), also known as coenzyme Q10 (Fig. 1A), is an FDA-approved antimicrobial drug used to treat pneumocystis pneumonia and to prevent and treat malaria caused by the parasites *Pnemocystis jirovecii* and *Plasmodium falciparum* and toxoplasmosis infections in immune-compromised HIV patients^1,2^. ATO exerts antiviral effects, inhibiting arboviruses^3^. ATO is the first clinically approved drug that targets *Plasmodium cytochrome bc*_1_ complex in mitochondria^4^. Also, ATO acts as a competitive inhibitor of mitochondrial complex III by displacing ubiquinol at the active site of the cytochrome *bc*_1_ complex, inhibiting mitochondrial respiration and mitochondrial membrane potential in parasites and killing them^5^.

Recently, ATO was repurposed to target mitochondrial complex III in breast cancer cells, and results show that ATO inhibits proliferation of breast cancer stem-like cells^6^. The *trans* form of ATO is significantly more potent than the *cis* form. A similar mechanism of action (mitochondrial complex III inhibition) was proposed in breast cancer cells^6^, although the affinity of ATO to mitochondrial cytochrome *bc*_1_ complex in mammalian cells is much lower than in parasites^7^. Targeting of ATO to mitochondrial complex III in ovarian cancer cells as a potential antitumor therapeutic strategy was proposed^8^. More recent studies show that ATO or ATO and proguanil (i.e., Malarone) exhibit antitumor activity both in animal models and in patients with acute myelogenous leukaemia and acute lymphocytic leukaemia^9^. Inhibition of tumour growth by ATO was attributed to inhibition of phosphorylation of signal transducer and activator of transcription 3 (STAT3)^10^. ATO has been shown to inhibit glioblastoma cell proliferation, and inhibition of STAT3 by ATO as a viable therapy for glioblastoma multiforme was proposed^10^. However, the ATO concentration in the brain was suggested to be too low to be chemotherapeutically effective.

We and others have previously shown that compared to their untargeted analogs, the triphenylphosphonium (TPP^+^)-conjugated derivatives are typically much more potent (> 50–100 times) in inhibiting tumour cell proliferation^11–14^. TPP^+^-conjugated mitochondria-targeted compounds are selectively taken into cancer cells at much higher levels due to an increased negative mitochondrial membrane potential^11,12^. The antiproliferative potency of TPP^+^-modified metformin, attributed to mitochondrial complex I inhibition, increased with increasing aliphatic side chain length^11,12^. Both metformin and mito-metformin analogs inhibit mitochondrial complex I-mediated respiration. Little or no information exists on TPP^ + ^-modified complex III inhibitors. Thus, we modified the structure of ATO, an established complex III inhibitor, and developed TPP^+^-conjugated ATO (Fig. 1A) and investigated their antiproliferative and oxidative phosphorylation (OXPHOS) inhibitory effects in cancer cells.

The potent inhibition of mitochondrial complex III may have implications in the maintenance of the immunosuppressive function of regulatory T (T_reg_) cells^15^. Although several relatively nontoxic mitochondrial complex I inhibitors^16^ exist (other than rotenone [Rot], which is toxic), antimycin A is one of the few complex III inhibitors presently available. Developing potent mitochondrial complex III inhibitors is timely because of their ability to suppress T_reg_ cells and enhance the levels of effector T (T_eff_) cells^15^.

In the present study, we show that mitochondria-targeted ATO (Mito-ATO) analogs (Fig. 1) are significantly more potent than ATO in inhibiting pancreatic cancer cell proliferation. Our results also show, for the first time, that conjugating ATO to TPP^+^ and increasing the aliphatic side chain length switches the molecular target in mitochondria from complex III/complex I to complex I for Mito-ATO analogs. As a result, Mito_12_-ATO and Mito_16_-ATO block mitochondrial respiration in pancreatic cancer cells by inhibiting only complex I and not complex III, whereas Mito_4_-ATO and Mito_10_-ATO inhibit oxygen consumption induced by both mitochondrial complex I and complex III. Potential implications of enhanced complex III inhibition induced by Mito_4_-ATO and Mito_12_-ATO in cancer immunosuppression are discussed.

## Results

### Mito-ATO analogs are more potent than ATO in inhibiting MiaPaCa-2 pancreatic cancer cell proliferation

Cell proliferation was monitored continuously in real time using an IncuCyte image analyser^11–13^ and both ATO and Mito-ATO analogs (Fig. 1A). Figure 1B shows the dose-dependent antiproliferative effects of ATO and Mito_10_-ATO. Mito_4_-ATO, Mito_12_-ATO, and Mito_16_-ATO also dose-dependently inhibited proliferation of MiaPaCa-2 pancreatic cancer cells. Mito-ATO analogs are more potent than ATO at inhibiting the proliferation of MiaPaCa-2 cells. Figure 1C shows the cell confluence (indicated by a dotted line) as a function of Mito-ATO concentration, and the half maximal inhibitory concentration (IC_50_) values of Mito_4_-ATO, Mito_10_-ATO, Mito_12_-ATO, and Mito_16_-ATO are 0.18 µM, 0.22 µM, 0.35 µM, and 3 µM, respectively. As compared with Mito-ATO analogs, ATO inhibited cell proliferation at much higher concentrations (IC_50_ = 18 µM) (Fig. 1C). These results suggest that attaching an aliphatic chain containing a TPP^+^ group to ATO greatly increases the antiproliferative potency. In control experiments, we used compounds (butyl-ATO and decyl-ATO) with an alkyl carbon–carbon side chain length similar to those of Mito_4_-ATO and Mito_10_-ATO but lacking the TPP^+^. As shown in Figure S3, both butyl-ATO and decyl-ATO devoid of TPP^+^ were much less effective than their corresponding Mito-ATO analogs.

### Inhibitory effects of Mito-ATO analogs on mitochondrial complex activities in MiaPaCa-2 cells

**Figure 2:**
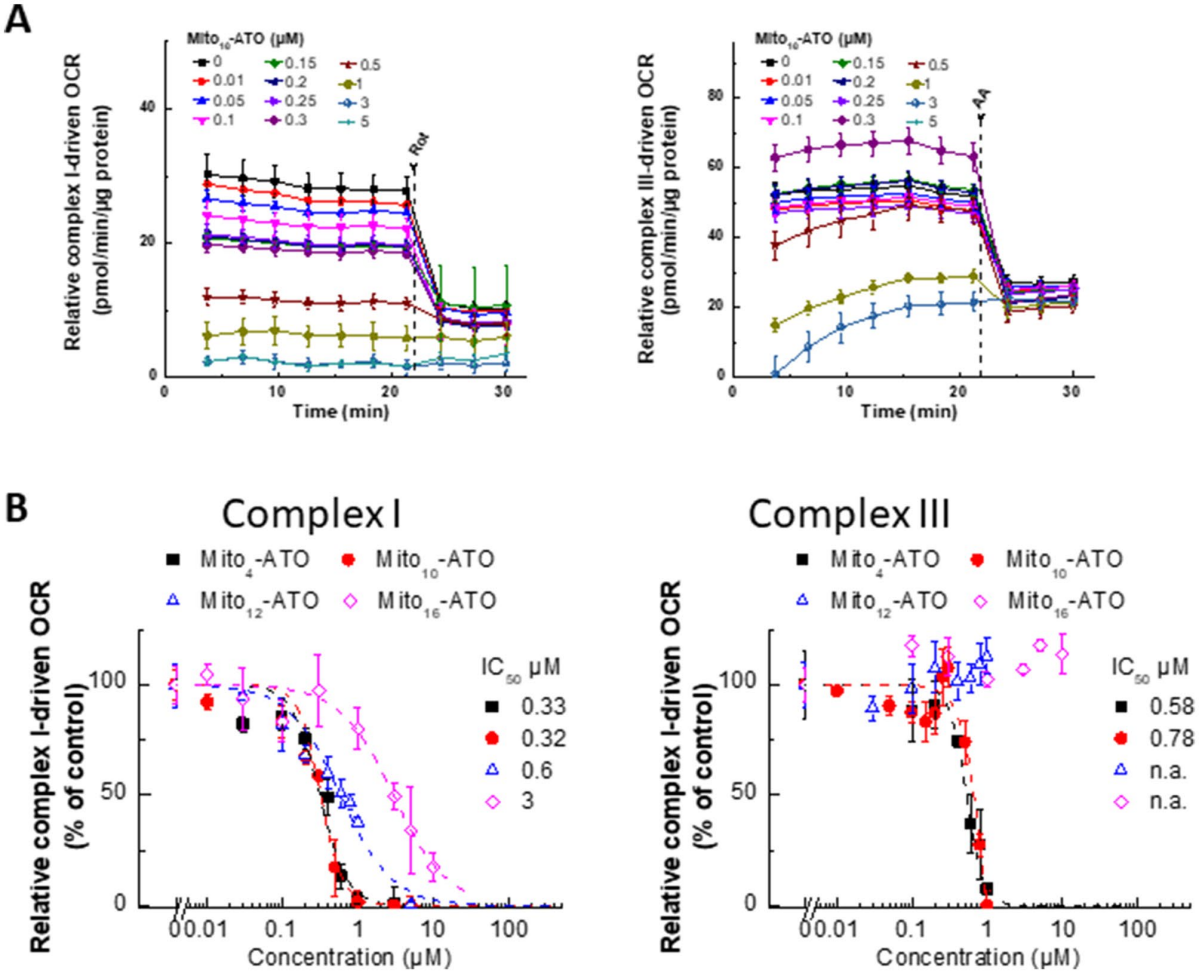
Effects of Mito-ATO analogs on the oxygen consumption induced by mitochondrial complexes I and III. (**A**) Dose-dependent effect of Mito_10_-ATO on complex I- and complex III-dependent oxygen consumption was measured in MiaPaCa-2 cells. MiaPaCa-2 cells were treated with Mito-ATO for 24 h. Relative complex I (1.5 mM malate, 10 mM pyruvate, 10 mM ADP)-driven OCR (left) and relative complex III (0.5 mM duroquinol, 10 mM ADP)-driven OCR (right) were monitored by XF-96 analyser. Either Rot (complex I inhibitor) or antimycin A (AA, complex III inhibitor) was acutely added and OCR assayed immediately. (**B**) The mitochondrial complex I (left)- and III (right)-driven OCR (calculated as Rot or AA inhibitable OCR) are plotted against the concentration of ATO and Mito-ATO analogs. Dashed lines represent the fitting curves used for determination of the IC_50_ values. (n.a., not applicable).

The mitochondrial complex activities were assessed by measuring the oxygen consumption rate (OCR) using the Seahorse technique^6,11–14^. MiaPaCa-2 cells were treated separately with Mito-ATO analogs at different concentrations for 24 h and OCR was measured. As shown in Fig. 2A, Mito_10_-ATO effectively inhibits complex III-induced oxygen consumption and, more importantly, Mito_10_-ATO caused a significantly greater inhibition of mitochondrial complex I-driven oxygen consumption. The IC_50_ value for Mito_10_-ATO to inhibit oxygen consumption by complex I is 0.32 µM, and the IC_50_ value for Mito_10_-ATO to inhibit oxygen consumption by complex III is 0.78 µM (Fig. 2B). Mito_4_-ATO and Mito_10_-ATO potently inhibit both mitochondrial complex I- and complex III-induced oxygen consumption. However, Mito_12_-ATO and Mito_16_-ATO did not inhibit complex III-induced oxygen consumption; they inhibited only the complex I-induced oxygen consumption (Fig. 2B). These results show that there is a shift in mitochondrial targeting of Mito-ATO analogs that is dependent on the alkyl side chain length attached to TPP^+^.

### Relative uptake of Mito_10_-ATO and ATO into MiaPaCa-2 and A549 cells

Next, we used the liquid chromatography with tandem mass spectrometry (LC–MS/MS) technique to investigate the relative uptake of Mito_10_-ATO and ATO in MiaPaCa-2 and A549 cells (Fig. S4). Cells were treated with Mito_10_-ATO (0.1 µM and 1 µM) or ATO (1–10 µM) for 1, 2, 4, and 24 h. As shown in Figure S4, there was an increase in cellular uptake of Mito_10_-ATO that accumulated inside the cells with time. Under the same treatment period with even with higher concentrations (up to 10 µM), ATO uptake was considerably lower and did not accumulate in cancer cells over a 24 h period.

### Inhibitory effects of Mito_10_-ATO on proliferation of breast and lung cancer cells

**Figure 3:**
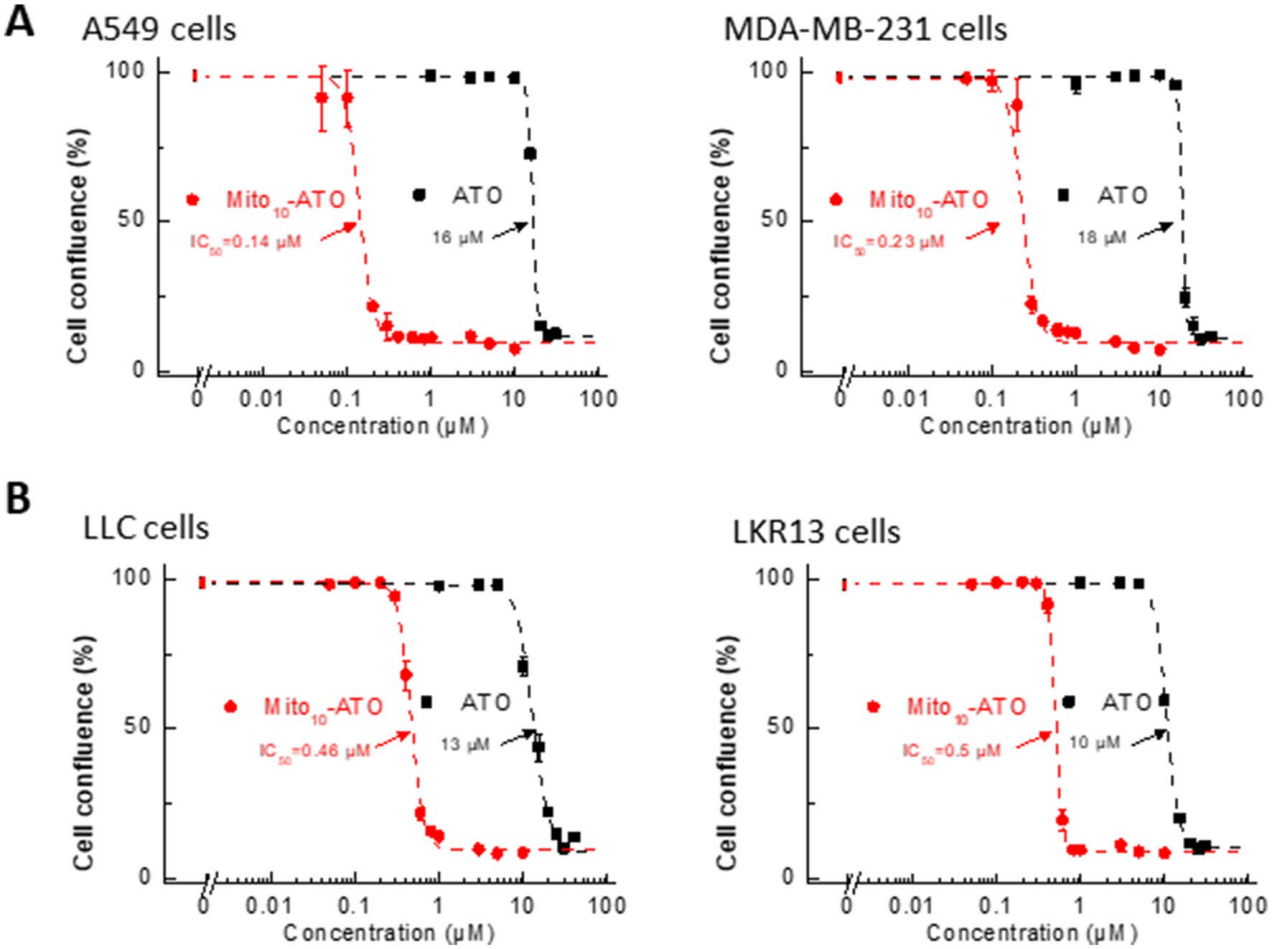
Effects of ATO and Mito-ATO on proliferation in human cancer cells and mouse cancer cells. A549 human lung and MDA-MB-231 human breast cancer cells (**A**) and LLC and LKR13 mouse lung cancer cells (**B**) were treated with ATO and Mito_10_-ATO. Cell proliferation was monitored in real-time with the continuous presence of indicated treatments until the end of each experiment. The cell confluence (as control groups reach 98% confluency) is plotted against concentration. Dashed lines represent the fitting curves used to determine the IC_50_ values as indicated.

**Figure 4:**
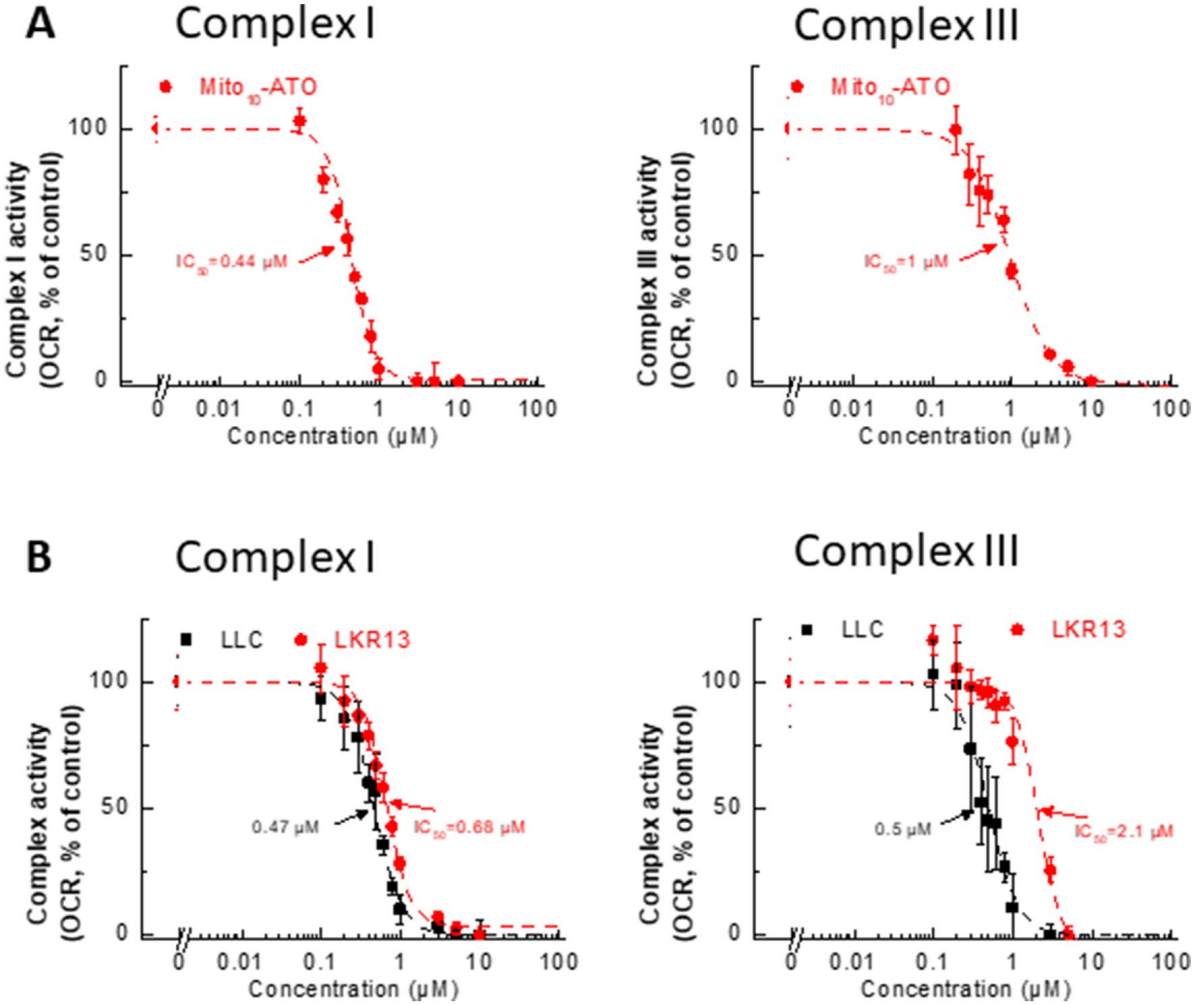
Effects of Mito_10_-ATO on the oxygen consumption induced by mitochondrial complexes I and III in human cancer cells and mouse cancer cells. A549 human lung cancer cells (**A**), and LLC and LKR13 mouse lung cancer cells (**B**) were treated with Mito_10_-ATO. The mitochondrial complex I (left) and complex III (right) -driven OCR (calculated as Rot or AA inhibitable OCR) are plotted against concentration of Mito_10_-ATO. Dashed lines represent the fitting curves used for determination of the IC_50_ values.

Previous reports provide evidence for enhanced uptake of several mitochondria-targeted cationic agents into cancer cells as compared with non-transformed control cells^11–14^. We examined the effect of ATO and Mito_10_-ATO on A549 human lung cancer cells and MDA-MB-231 human breast cancer cells (Fig. 3A) and on LLC and LKR13 mouse lung cancer cells (Fig. 3B). Mito_10_-ATO was effective at halting cell proliferation in A549 and MDA-MB-231 human cancer cells and in LLC and LKR13 mouse lung cancer cells. As shown in Fig. 3, Mito_10_-ATO also was more potent than ATO in inhibiting cancer cell proliferation. Mito_10_-ATO inhibited both mitochondrial complex I- and III-induced oxygen consumption in these cells (Fig. 4). These results demonstrate that the enhanced antiproliferative potency of Mito-ATO analogs is not restricted to a single cancer cell type and is broadly applicable to several cancer cells.

### Immunomodulatory effects of Mito_4_-ATO, Mito_10_-ATO, and Mito_12_-ATO

**Figure 5:**
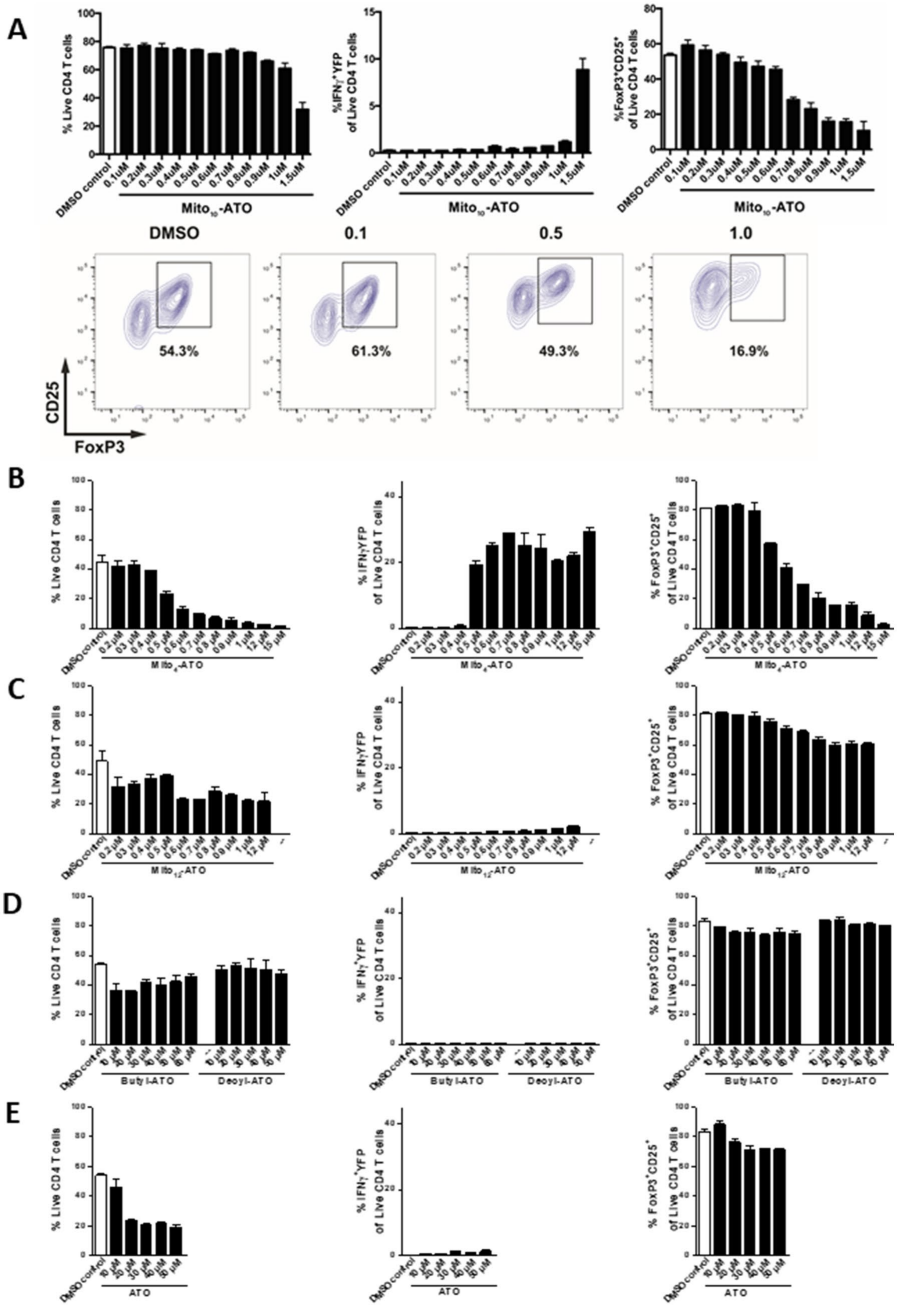
In vitro differentiation of CD4^+^ T regulatory cells under varying concentrations of Mito-ATOs and controls. After six days of culture, cells were stained for flow cytometry analysis. (left) Live/dead staining to assess the percentage of live CD4 + T cells within the lymphocyte gate. (middle) T_eff_ cell function is shown as the frequency of IFNγ-YFP positive cells within the live CD4^+^ T cells. (right) The percentage of T_reg_ cells is shown as the frequency of FoxP3^+^CD25^+^ cells within the live CD4^+^ T cells. (**A**, bottom) Representative plot using FlowJo Software, effects of Mito_10_-ATO on T_reg_ (FoxP3^+^CD25^+^) cells within the live CD4^+^ T cell gate (contour plots depict gating corresponding to panel **A**, upper right)^39^. Mito_4_-ATO (**B**) effectively suppressed T_reg_ while Mito_12_-ATO (**C**), Butyl-ATO (**D**), Decyl-ATO (**D**), and ATO (**E**) did not suppress T_reg_ cells.

Recent reports indicate that selective targeting and inhibiting of mitochondrial complex III mitigate and reverse immunosuppression by T_reg_ cells, promoting the function of T_eff_ cells^15,17^. To investigate the effects of Mito_4_-ATO, Mito_10_-ATO, and Mito_12_-ATO on T_eff_ *versus* T_reg_ cells, activated CD4^+^ T cells were isolated from SMARTA triple reporter mice, activated, and cultured in vitro with TGFβ (5 ng/mL) and IL-2 (100 ug/mL), as described in the Materials and Methods section. The CD4^+^ T cells were treated with ATO and Mito-ATO analogs at varying concentrations (Fig. 5). After six days, cells were stained to assess viability, phenotype, and function using flow cytometry. Results demonstrate that Mito_4_-ATO (Fig. 5B) and Mito_10_-ATO (Fig. 5A) inhibited Foxp3^+^ T_reg_ differentiation and/or survival and promoted T_eff_ cell IFNγ production in a dose-dependent manner. In contrast, Mito_12_-ATO did not appreciably inhibit T_reg_ differentiation (Fig. 5C). Mito_4_-ATO and Mito_10_-ATO potently inhibited mitochondrial complex I- and complex III-driven oxygen consumption. Mito_12_-ATO strongly inhibited oxygen consumption by complex I- but not complex III-driven oxygen consumption. Thus, it is plausible that inhibition of T_reg_ and stimulation of T_eff_ response by Mito_4_-ATO and Mito_10_-ATO are mediated by their increased potency to target mitochondrial complex III. Furthermore, ATO, butyl-ATO, and decyl-ATO (up to 60 µM) did not inhibit T_reg_ differentiation and/or survival (Fig. 5D, E).

## Discussion

### The relative hydrophobicity of ATO, Mito-ATO, and alkyl-ATO

The lipophilicity of ATO was attributed to its stabilization in the hydrophobic pocket of the cytochrome *bc*_1_ complex^5^. To assess the relative hydrophobicity of ATO and Mito-ATO analogs, we calculated the octanol/water partition coefficients (log P) using a QSAR analysis and rational drug design as a measure of molecular hydrophobicity (Table 1). This method also uses a consensus model built using the ChemAxon software (San Diego, CA)^18,19^. Table 1 lists the log P values along with the calculated regions of the relative hydrophilic and hydrophobic regions. As shown in Table 1, there was a significant increase in the hydrophobicity of Mito-ATO (as compared with ATO) with the increasing alkyl side chain length (from C-4 to C-16).

**Table 1:**
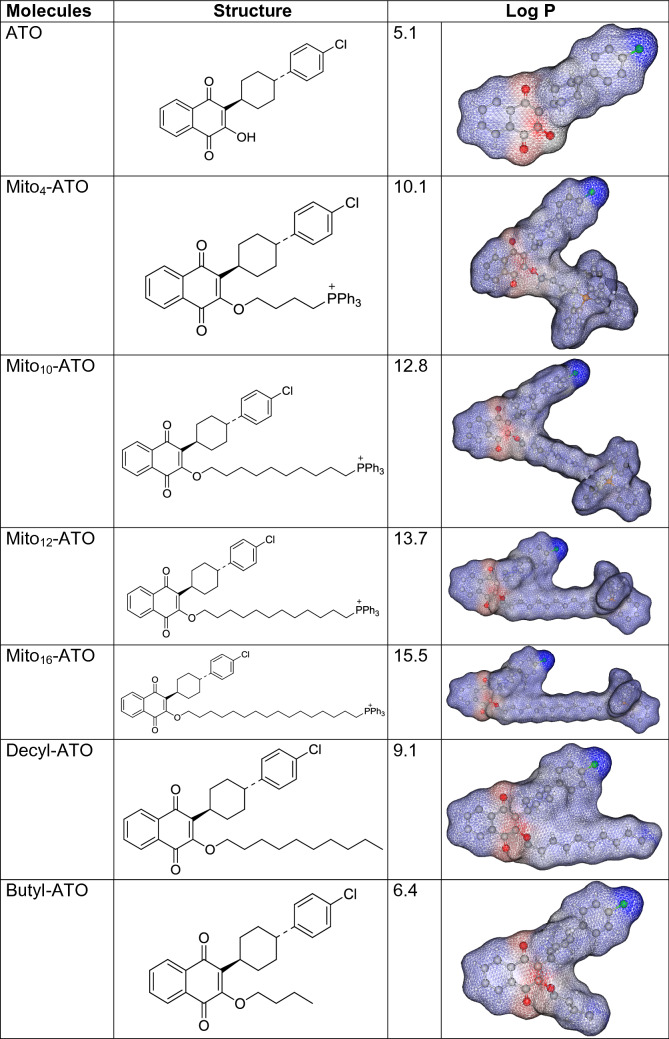
Calculated partition coefficients and relative hydrophobic regions in ATO and Mito-ATO analogs.

### Inhibition of cytochrome *bc*_1_ by ATO: Stabilizing molecular interactions at the active site

**Figure 6:**
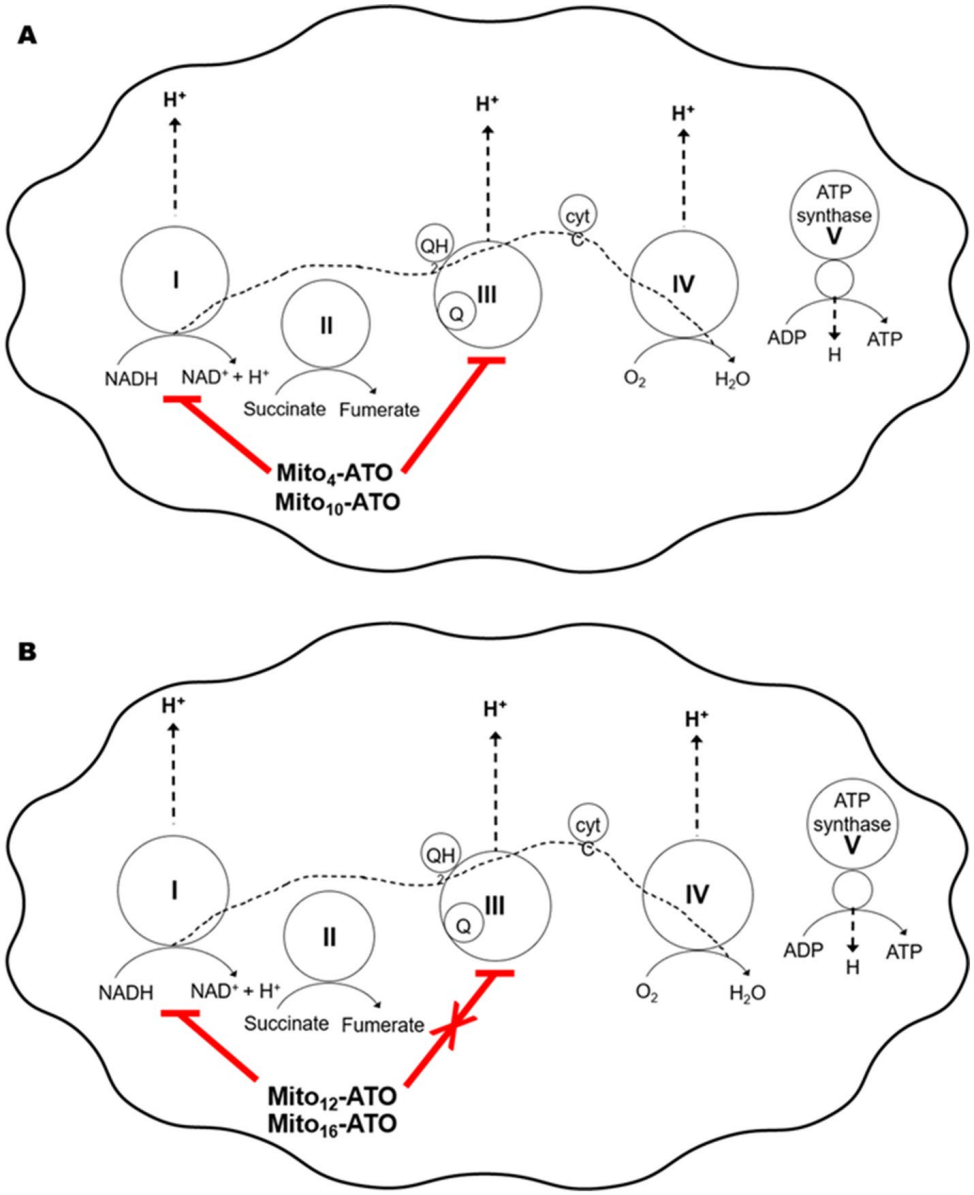
Mitochondrial targeting of Mito-ATOs with varying side chain lengths. This figure illustrates (**A**) the dual mitochondrial targeting (complex I and complex III) of Mito_4_-ATO and Mito_10_-ATO and (**B**) a shift in mitochondrial targeting from complex III to complex I for Mito_12_-ATO and Mito_16_-ATO in MiaPaCa-2 cells.

Previous studies showed that ATO binds to the mitochondrial cytochrome *bc*_1_ complex (ubiquinol cytochrome *c* oxidoreductase or complex III) and inhibits its activity^5,20^. We report here that structural modification of ATO by attachment of TPP^+^ to ATO (Mito-ATOs) greatly inhibits tumour cell proliferation. Both Mito_4_-ATO and Mito_10_-ATO potently inhibit oxygen consumption by complex I and complex III (Fig. 6A). Surprisingly, Mito_12_-ATO (with a 12-carbon side chain) and Mito_16_-ATO (with a 16-carbon side chain) only inhibited complex I- but not complex III-driven oxygen consumption (Fig. 6B). Conceivably, the lack of effect of Mito_12_-ATO and Mito_16_-ATO on oxygen consumption by complex III suggests that Mito_12_-ATO and Mito_16_-ATO do not target the Qo site of the cytochrome *bc*_1_ complex.

ATO is structurally similar to Q and acts as a competitive inhibitor of Q. Modelling and energy minimization studies show that ATO docks into the Qo active site stabilized by hydrophobic and hydrogen bonding interactions with the Rieske protein and the cytochromes^5,21^. Q is reduced to ubiquinol (QH2) by complexes I and II and oxidized to Q at the catalytic site of complex III. Based on published reports on the molecular basis of the antimalarial action of ATO^5^, a similar mechanism is proposed for ATO inhibition of respiration of cancer cells. ATO acts as a competitive Qo site-specific inhibitor of cytochrome *bc*_1_ complex oriented between the [2Fe–2S] cluster of the Rieske protein. ATO is stabilized in this pocket through the polarized hydrogen bonding interaction between the oxygen atom (O) of the ionized form and the protonated nitrogen atom of the His181 side chain of the Rieske protein.

The evidence for the binding of ATO to the Rieske iron-sulphur centre came from electron paramagnetic resonance (EPR) spectroscopy and circular dichroism spectroscopy studies of the purified cytochrome *bc*_1_ complex from the yeast *Saccharomyces cerevisiae*^4^. The addition of ATO shifted the ascorbate-reduced Rieske centre signals (at gz and gx to a higher field and the gy component to a lower field). The extent of the magnetic field shifts in the EPR spectra of the reduced Rieske Fe-S centre indicates a change in the electronic environment of the 2Fe-2S cluster due to ATO binding^4^. The low-temperature EPR of the yeast system may be suitable for investigating the effect of alkyl side chain length in Mito-ATO (e.g., Mito_4_-ATO, Mito_10_-ATO, Mito_12_-ATO, or Mito_16_-ATO) and the Rieske iron-sulphur cluster.

It is plausible that, depending on the length of the aliphatic substituent attached to the TPP^+^ group in Mito-ATO, some Mito-ATO analogs (Mito_4_-ATO or Mito_10_-ATO) may be stabilized at the cytochrome *bc*_1_ pocket by several hydrophobic/aromatic interactions between the ATO moiety and the amino acid residues within the binding site. Mito-ATO is much more hydrophobic than ATO. It accumulates into cancer cell mitochondria more effectively than ATO and inhibits mitochondrial respiration and cancer cell proliferation more potently than ATO. Results using Mito_12_-ATO indicate that it does not inhibit complex III-dependent oxygen consumption. However, Mito_12_-ATO potently inhibits complex I-driven oxygen consumption (Fig. 6). This, combined with the finding that Mito_12_-ATO does not inhibit T_reg_ cells, implies that the alkyl side chain length in Mito-ATO plays an important role in mitochondrial targeting and immunoregulatory effects^22^.

### Cancer immunosuppression and Mito-ATO

The number of T_reg_ cells is reportedly increased in the microenvironments of most cancers (e.g., pancreatic cancer, lung cancer)^23,24^. There is negative correlation between T_reg_ levels and survival in cancer patients^25^. T_reg_ cells suppress antitumor immunity, thereby hampering immunotherapy^26^. Drug therapy targeting T_reg_ cells is emerging as a promising antitumor approach^27,28^. Emerging research shows that mitochondrial respiratory chain activity, particularly mitochondrial complex III activity, is crucial for preserving the antitumor function of T_reg_ cells^29^. Alternatively, T_reg_ cells devoid of complex III had decreased immunoregulatory function^15^. It is conceivable that Mito-ATO analogs with the appropriate substituents and aliphatic side chain lengths will be able to inhibit T_reg_ cell respiration and activate cancer immunotherapy.

Clearly, the discovery that increasing alkyl chain length in Mito-ATO, a new class of small-molecule OXPHOS inhibitors, changes the mitochondrial respiratory complex target is significant in corroborating the role of complex III-dependent metabolic alterations (i.e., accumulation of oncometabolite 2-hydroxyglutarate [2-HG] and succinate) in repressing alpha-ketoglutarate-dependent demethylases and DNA hypermethylation^15^. Previously, it was shown that loss or inhibition of mitochondrial complex III in cancer cells results in increased levels of 2-HG and succinate^15,17^. Antimycin A increased the levels of succinate and 2-HG in T_reg_ cells^15^. Our initial attempts to measure mitochondrial complex activities, using the Seahorse technique, in intact and permeabilized T cells were not successful. Several experimental conditions (e.g., cell concentration, permeabilizing conditions) need to be optimized before reliable oxygen consumption rate measurements can be made. To further corroborate the role of mitochondrial complex III, it would be of interest to compare the effects of ATO, Mito_4_-ATO, Mito_10_-ATO, Mito_12_-ATO, and Mito_16_-ATO on inducing the transcriptional programs responsible for metabolic alterations in cancer cells and immune cells^15,17^.

In this study, we showed that TPP^+^-conjugated ATO analogs (Mito_4_-ATO, Mito_10_-ATO, Mito_12_-ATO, and Mito_16_-ATO) are considerably more potent than the parent drug, ATO, at inhibiting the proliferation of pancreatic and other cancer cell types. Mito_4_-ATO and Mito_10_-ATO but not Mito_12_-ATO suppress T_reg_ function. The molecular targets of Mito-ATO analogs in the mitochondrial respiratory chain are different depending on the alkyl side chain length. Mito_4_-ATO and Mito_10_-ATO inhibit mitochondrial oxygen consumption by complex I and complex III, whereas Mito_12_-ATO and Mito_16_-ATO inhibit only the complex I-induced oxygen consumption. Interestingly, the alkyl side chain length in Mito-ATOs influences their mitochondrial targeting and inhibition of oxygen consumption.

## Materials and methods

### General

All chemicals and organic solvents were commercially available and were used as supplied. The reactions were monitored by thin layer chromatography using silica gel Merck ^60^F254. Crude materials were purified by flash chromatography on Merck silica gel 60 (0.040–0.063 mm). ^31^P NMR, ^1^H NMR and ^13^C NMR spectra were recorded at 400 and 75 MHz spectrometers, respectively. ^1^H NMR spectra were recorded using a Bruker DPX AVANCE 400 spectrometer equipped with a quattro nucleus probe. Chemical shifts (δ) are reported in ppm and *J* values in Hertz.

Syntheses and nuclear magnetic resonance (NMR) data of appropriate ATO controls lacking the TPP^+^ group are given in Figs. S1 and S2 in the Supplementary Materials.

### Synthesis of Mito-ATO analogs

The mitochondria-targeted analogs of ATO (Mito_n_-ATO) were prepared by reacting the appropriates bromoalkyl-triphenylphosphonium bromides with ATO in the presence of potassium carbonate in dimethylformamide (DMF) (Fig. S1). In addition, the untargeted ATO derivatives (ATO-C_n_) were prepared by adapting the procedure to the corresponding alkyl bromides (Fig. S1).

### Synthesis of Mito_4_-ATO

Mito_4_-ATO was prepared by reacting (4-bromobutyl)-triphenylphosphonium bromide with ATO in the presence of potassium carbonate in DMF (Fig. S1). Briefly, (4-bromobutyl)-triphenylphosphonium bromide (0.39 g, 0.81 mmol) was added to a mixture of ATO (0.3 g, 0.82 mmol) and potassium carbonate (0.15 g, 0.82 mmol) in DMF. The mixture was stirred at 70 °C for 9 h. CH_2_Cl_2_ was then added to the mixture followed by the addition of water (20 mL). The organic layer was washed twice with water and dried over Na_2_SO_4_. The solvent was removed under reduced pressure. Diethyl ether was added to the mixture to precipitate out the compound that was purified by flash chromatography (CH_2_Cl_2_/EtOH, 9:1), yielding the product, Mito_4_-ATO (0.47 g, 75% yield).

The HRMS calculated and found values are C_44_H_41_ClO_3_P^+^ [M^+^] 683.2476 and 683.2479. ^31^P NMR (400.13 MHz, CDCl_3_) δ 24.66. ^1^H NMR (400.13 MHz, CDCl_3_), δ 8.06–8.03 (1H, m), 7.95–7.93 (1H, m), 7.92–7.85 (6H, m), 7.79–7.72 (3H, m), 7.71–7.63 (8H, m), 7.25–7.21 (2H, m), 7.15–7.12 (2H, m), 4.26 (2H, t, *J* = 5.6), 4.11–4.02 (2H, m), 3.13–3.03 (1H, m), 2.58–2.48 (1H, m), 2.37–2.28 (2H, m), 2.10–1.98 (4H, m), 1.92–1.88 (2H, m), 1.65 (2H, dd, *J* = 12.7, 2.7), 1.54–1.39 (2H, m). ^13^C NMR (75 MHz, CDCl_3_) δ 185.4, 181.7, 157.7, 145.7, 140.0, 134.9, 134.8, 133.8, 133.77, 133.68, 133.2, 132.3, 131.4, 131.3, 130.5, 130.3, 128.3, 128.2, 118.8, 117.9, 72.4, 43.1, 35.5, 34.3, 30.12 (d, 17.6), 30.1, 30.0, 22.2 (d, *J* = 50.6), 19.1 (d, *J* = 3.7).

NMR spectra and related parameters are included in Fig. S2.

### Synthesis of Mito_10_-ATO

Mito_10_-ATO was prepared by reacting (10-bromodecyl)-triphenylphosphonium bromide with ATO in the presence of potassium carbonate in DMF (Fig. S1). Briefly, (10-bromodecyl)-triphenylphosphonium bromide (1.1 g, 1.9 mmol) was added to a mixture of ATO (0.73 g, 1.9 mmol) and potassium carbonate (0.3 g, 2.1 mmol) in DMF (4 mL). The mixture was stirred at 70 °C for 9 h. Methylene chloride (CH_2_Cl_2_) was added to the mixture, and then water (20 mL) was added. The organic layer was washed twice with water and dried over anhydrous sodium sulphate. The excess solvent was removed under reduced pressure. Diethyl ether was subsequently added to the mixture to precipitate the compound. Purification by flash chromatography, using a mixture of methylene chloride and ethanol (9:1), yielded the desired compound, Mito_10_-ATO (1 g, 59% yield).

The high-resolution mass spectral (HRMS) calculated for Mito_10_-ATO C_50_H_53_ClO_3_P^+^ [M]^+^ 767.3415, found, 767.3420. ^31^P NMR (400.13 MHz, CDCl_3_) δ 24.48. ^1^H NMR (400.13 MHz, CDCl_3_, δ 8.08–7.98 (2H, m), 7.90–7.82 (6H, m), 7.81–7.75 (3H, m), 7.73–7.64 (8H, m), 7.25–7.23 (2H, m), 7.18–7.16 (2H, m), 4.30 (2H, t, *J* = 6.6), 3.88–3.77 (2H, m), 3.25–3.15 (1H, m), 2.66–2.55 (1H, m), 2.24–2.11 (2H, m), 2.01–1.92 (2H, m), 1.86–1.77 (2H, m), 1.76–1.68 (4H, m), 1.60–1.40 (6H, m), 1.38–1.25 (8H, m). ^13^C NMR (75 MHz, CDCl_3_) δ 185.5, 181.9, 158.0, 146.0, 138.6, 134.9, 134.8, 133.8, 133.72, 133.67, 133.1, 132.4, 131.5, 131.4, 130.5, 130.4, 128.4, 128.2, 126.3, 125.9, 118.9, 118.1, 74.0, 43.3, 35.4, 34.5, 30.4 (d, *J* = 14.0), 29.9, 29.5, 29.3, 29.2, 25.9, 22.8 (d, *J* = 49.1), 22.7 (d, *J* = 4.4).

NMR spectra and related parameters are given in Fig. S2.

### Synthesis of Mito_12_-ATO

Mito_12_-ATO was prepared by reacting (12-bromodecyl)-triphenylphosphonium bromide with ATO in the presence of potassium carbonate in DMF (Fig. S1) as follows: (12-bromododecyl)-triphenylphosphonium bromide (0.6 g, 1.0 mmol) was added to a mixture of ATO (0.45 g, 1.2 mmol) and potassium carbonate (0.17 g, 1.2 mmol) in DMF. The mixture was stirred at 70 °C for 7 h. CH_2_Cl_2_ was added to the mixture followed by water (20 mL). The organic layer was washed twice with water and dried over Na_2_SO_4_. The solvent was removed under reduced pressure. Diethyl ether was then added to precipitate out the compound that was purified by flash chromatography (CH_2_Cl_2_/EtOH, 9:1) yielding the product, Mito_12_-ATO (0.35 g, 35% yield).

HRMS calculated for Mito_12_-ATO C_52_H_57_ClO_3_P^+^ [M]^+^ 795.3728, found, 795.3729.

^31^P NMR (400.13 MHz, CDCl_3_) δ 24.44. ^1^H NMR (400.13 MHz, CDCl_3_), δ 8.07–7.97 (2H, m), 7.87–7.74 (9H, m), 7.72–7.63 (8H, m), 7.24–7.20 (2H, m), 7.18–7.13 (2H, m), 4.30 (2H, t, *J* = 6.6), 3.82–3.72 (2H, m), 3.25–3.13 (1H, m), 2.65–2.55 (1H, m), 2.23–2.09 (2H, m), 1.99–1.92 (2H, m), 1.84–1.67 (7H, m), 1.63–1.42 (8H, m), 1.37–1.19 (9H, m). ^13^C NMR (75 MHz, CDCl_3_) δ 185.5, 181.9, 158.1, 146.0, 138.6, 134.92, 134.89, 133.7, 133.6, 133.1, 132.4, 131.5, 131.4, 130.5, 130.4, 128.4, 128.2, 126.3, 125.9, 118.9, 118.1, 74.1, 43.3, 35.4, 34.5, 30.5, 30.4, 30.3 (d, *J* = 16.1), 29.9, 29.6, 29.5, 29.3, 29.24, 29.21, 25.9, 22.8 (d, *J* = 49.2), 22.4 (d, *J* = 4.4).

The NMR spectra and related parameters are given in Fig. S2.

### Synthesis of Mito_16_-ATO

Mito_16_-ATO was prepared by reacting (16-bromohexadecyl)-triphenylphosphonium bromide with ATO in the presence of potassium carbonate in DMF as follows: (16-bromohexadecyl)-triphenylphosphonium bromide (0.2 g, 0.34 mmol) was added to a mixture of ATO (0.13 g, 0.37 mmol) and potassium carbonate (0.05 g, 0.37 mmol) in DMF (2 mL). The mixture was stirred at 70 °C for 7 h. CH_2_Cl_2_ was added to the mixture as well as water (20 mL). The organic layer was washed twice with water and dried over Na_2_SO_4_. The solvent was removed under reduced pressure. Diethyl ether was the added to the mixture to precipitate out the compound that was purified by flash chromatography (CH_2_Cl_2_/EtOH, 9:1), yielding the product, Mito_16_-ATO (0.16 g, 54% yield).

HRMS calculated for Mito_16_-ATO C_56_H_65_ClO_3_P^+^ [M]^+^ 851.4354, found, 851.4360.

^31^P NMR (400.13 MHz, CDCl_3_) δ 23.39. ^1^H NMR (400.13 MHz, CDCl_3_), δ 8.00–7.93 (2H, m), 7.81–7.59 (17H, m), 7.21–7.16 (2H, m), 7.11–7.09 (2H, m), 4.26 (2H, t, *J* = 6.7), 3.75–3.63 (2H, m), 3.20–3.08 (1H, m), 2.60–2.48 (1H, m), 2.17–2.03 (2H, m), 1.94–1.85 (2H, m), 1.80–1.73 (2H, m), 1.70–1.63 (2H, m), 1.68–1.38 (8H, m), 1.33–1.06 (20H, m). ^13^C NMR (75 MHz, CDCl_3_) δ 185.54, 181.8, 158.0, 146.0, 138.5, 134.94, 134.91, 133.7, 133.6, 133.0, 132.3, 131.3, 131.4, 130.5, 130.4, 128.3, 128.1, 126.2, 125.9, 118.8, 118.0, 74.0, 43.3, 35.5, 34.5, 30.5, 30.4, 30.3, 29.9, 29.6, 29.59, 29.56, 29.52, 29.4, 29.3, 29.2, 29.1, 25.9, 22.8 (d, *J* = 49.9), 22.6 (d, *J* = 4.4).

The NMR spectra and related parameters are given in Fig. S2.

### Cell culture

The following cell lines were obtained from the American Type Culture Collection (Manassas, VA), where they were regularly authenticated: MiaPaCa-2 (ATCC#CRL-1420, human pancreatic cancer), MDA-MB-231 (ATCC#HTB-26, human breast cancer), A549 (ATCC#CCL-185, human lung cancer), LLC (ATCC#CLR-1642, mouse lung cancer). LKR13 mouse lung cancer cells were a gift from Dr. Jonathan M. Kurie (MD Anderson Cancer Center, Houston, TX)^30^. All cell lines were grown at 37 °C in 5% CO_2_. MiaPaCa-2 and MDA-MB-231 cells were maintained in DMEM medium (Thermo Fisher Scientific, #11965) supplemented with 10% foetal bovine serum. A549, LLC, and LKR13 cells were maintained in RPMI 1640 medium (Thermo Fisher Scientific, #11875), supplemented with 10% foetal bovine serum. All cells were stored in liquid nitrogen and used within 20 passages after thawing.

### Cell proliferation measurements

The IncuCyte Live-Cell Imaging system (IncuCyte Essen Bioscience Inc., Ann Arbor, MI) was used to monitor cell proliferation^11–13^. As shown in previous publications^11,12^, this imaging system is probe-free and noninvasive, and enables continuous monitoring of cell confluence over several days. The increase in the percentage of cell confluence was used as a surrogate marker of cell proliferation. In a 96-well plate, cells were plated at 1,000 cells per well in triplicates and left to adhere overnight. Cells were then treated with ATO, Mito-ATO analogs, and appropriate controls, and the cell confluency was recorded over several days in the IncuCyte S3 system.

### Mitochondrial function measurements

Mitochondrial bioenergetic function was measured in real time using the Seahorse XF 96 Extracellular Flux Analyzer (Agilent, North Billerica, MA)^11–13,22^. The OCR-based assessment of mitochondrial complex activities was carried out on acutely permeabilized cells in the presence of different mitochondrial substrates, i.e., pyruvate/malate, for complex I and duroquinol for complex III^11,14,31,32^. Rot, malonate, and antimycin A (Sigma-Aldrich, St. Louis, MO) were used as specific inhibitors of mitochondrial complexes I, II, and III, respectively. Briefly, cells that were intact after treatments were immediately permeabilized using the Seahorse XF Plasma Membrane Permeabilizer (Agilent). The mitochondrial complex I-driven OCR was assayed in mannitol and sucrose buffer^31^ containing 10 mM pyruvate and 1.5 mM malate (substrates for complex I) and 10 mM malonate (which inhibits complex II activities). The mitochondrial complex III-driven OCR was assayed in mannitol and sucrose buffer containing 0.5 mM duroquinol (substrate for complex III) and 1 µM Rot and 10 mM malonate (which inhibit both complex I and II activities). The mitochondrial complex-dependent oxygen consumption (calculated as Rot- or antimycin A-inhibitable OCR equals basal OCR less OCR after Rot or antimycin A injection) was plotted against concentrations to determine the IC_50_ values.

### Immunosuppression measurements

#### Mice

SMARTA triple reporter mice were generated in the following manner. First, IL-10 and IL-21 double-reporter mice^33^ were generated by cross-breeding IL-21tRFP mice^34,35^ with 10 BiT mice (kindly provided by Dr. Casey Weaver, University of Alabama at Birmingham). Double reporter mice were then crossed with GREAT (interferon-gamma reporter with endogenous polyA transcript) mice^36^ from Jackson Laboratory (Stock No. 017581). These triple-reporter mice were then crossed with SMARTA mice^37^ (kindly provided by Dr. Dorian McGavern, National Institutes of Health). Mice were bred and maintained in a closed breeding facility, and mouse handling conformed to the requirements of the Medical College of Wisconsin Institutional Animal Care and Use Committee guidelines. All experimental protocols were approved by the Medical College of Wisconsin Institutional Animal Care and Use Committee.

#### Cell culture

To differentiate CD4^+^ T cells into a T regulatory cell phenotype, splenocytes from SMARTA triple-reporter mice were processed and the red blood cell lysed using an ACK (ammonium-chloride-potassium) lysis buffer. The cells were then activated with 1 μg/mL GP_61–80_ peptide (GenScript, Piscataway, NJ) and 5 ng/mL TGF-β1 (Shenandoah Biotechnology, Inc., Warwick, PA). After one day of initial skewing, 100 μg/mL IL-2 along with ATO or Mito-ATO analogs of varying concentrations were added to the culture. Cells were cultured for six days and split once cells reached confluency; cells were replenished with IL-2 and compound accordingly. After six days in culture, cells were stained to assess the viability and phenotypic analysis via flow cytometry. LIVE/DEAD fixable violet or aqua dead cell stain (Invitrogen, Carlsbad, CA) was used to assess cell viability. The following antibodies were used for flow cytometry staining: PercP anti-mouse CD4 (clone: GK1.5; BioLegend, San Diego, CA), APC/Cy7 anti-mouse CD25 (clone: PC61; BioLegend), and PE anti-mouse FOXP3 (clone: FJK-16S; eBioscience, San Diego, CA). Flow cytometry data were acquired using a BD LSRII (BD Biosciences, CA) flow cytometer and analysed using FlowJo (Treestar, Inc., Ashland, OR).

### Uptake of ATO and Mito_10_-ATO into MiaPaCa-2 and A549 cells

Intracellular levels of ATO and Mito_10_-ATO analogs were quantitated by LC–MS/MS^11,12^. Cells (1 × 10^6^ per dish) were grown in 10-cm dishes and incubated with ATO and Mito_10_-ATO for 24 h in full culture media. The compounds were extracted according to the published experimental protocol^11,12^. Briefly, cells were washed twice with ice-cold DPBS (Dulbecco’s Phosphate Buffered Saline) and harvested. The cell pellet was immediately frozen in liquid nitrogen and stored at − 80 °C. For the extraction, the pellet was homogenized in 200 µl DPBS. A total of 2 µl were taken for the protein assay and 180 µl were extracted twice with a dichloromethane:methanol (2:1) mixture. The organic layers were combined and dried using a SpeedVac concentrator (Thermo Fisher Scientific, Waltham MA). The dry residue was dissolved in 100 µl of ice-cold methanol and taken for LC–MS/MS analysis. LC–MS/MS analyses were performed using a Kinetex Phenyl-Hexyl column (50 mm × 2.1 mm, 1.7 µm; Phenomenex, Torrance, CA) equilibrated with a water:acetonitrile mixture (4:1) containing 0.1% formic acid. Compounds were eluted by increasing the content of acetonitrile from 20 to 100% over 4 min and detected using the MRM (multiple reaction monitoring) mode. The protein concentrations were determined by the standard Bradford method (Bio-Rad, Hercules, CA). In brief, 2 µl of sample was added to 198 µl of preformulated Coomassie Brilliant Blue G-250 assay reagent (Bio-Rad), and the resultant blue colour was measured at 595 nm. The total protein level of the sample used to perform the extraction was calculated as milligrams of protein per sample. Intracellular concentrations (nmol/mg protein) of ATO and Mito_10_-ATO were calculated based on the LC–MS/MS standard curve, and the results were normalized to the total protein level.

## Supplementary information

Supplementary information.
