# Supplementary material for: Potent inhibition of tumour cell proliferation and immunoregulatory function by mitochondria-targeted atovaquone
Source: Sci Rep. 2020 Oct 21;10:17872. doi: 10.1038/s41598-020-74808-0 (PMC7578061; doi:10.1038/s41598-020-74808-0)
Supplement: Supplementary file 1 — Supplementary information. [file 41598_2020_74808_MOESM1_ESM.docx]

**Supplementary Materials**

**Potent inhibition of tumour cell proliferation and immunoregulatory function by mitochondria-targeted atovaquone**

Gang Cheng^1,2^, Micael Hardy^6^, Paytsar Topchyan^3,7^, Ryan Zander^3,7^, Peter Volberding^3,7^, Weiguo Cui^3,7^, and Balaraman Kalyanaraman^1,2,4,5^

^1^Department of Biophysics, ^2^Free Radical Research Center, ^3^Microbiology & Immunology, ^4^Cancer Center, ^5^Center for Disease Prevention Research, Medical College of Wisconsin, 8701 Watertown Plank Road, Milwaukee, WI 53226, United States

^6^Aix Marseille Univ, CNRS, ICR, UMR 7273, Marseille 13013, France

^7^Versiti Blood Research Institute, 8733 Watertown Plank Road, Milwaukee, WI 53226, United States

**Corresponding author:** Balaraman Kalyanaraman, Department of Biophysics, Medical College of Wisconsin, 8701 Watertown Plank Road, Milwaukee, WI 53226, United States; phone: 414-955-4000; fax: 414-955-6512; email: balarama@mcw.edu

**Synthesis of ATO-C_10_**

*Decyl-ATO (*ATO-C_10_*)* was prepared by reacting bromodecane with ATO in the presence of potassium carbonate in DMF (Figure S1).

Bromodecane (0.24 g, 1.1 mmol) was added to a mixture of ATO (0.4 g, 1.1 mmol) and potassium carbonate (0.15 g, 1.1 mmol) in DMF (3 mL). The mixture was stirred at 70°C for 7 h. Diethyl ether was then added to the mixture as well as water (20 mL). The organic layer was washed twice with water and dried over Na_2_SO_4_. The solvent was removed under reduced pressure. The compound was purified by flash chromatography (hexane/ethyl acetate, 95:5), yielding the final product (0.45 g, 81% yield).

HRMS calculated for ATO-C_10_ C_32_H_39_ClO_3_ [MH]^+^ 507.2660, found, 507.2659.

^1^H NMR (400.13 MHz, CDCl_3_), δ 8.00-7.94 (2H, m), 7.65-7.58 (2H, m), 7.21-7.18 (2H, m), 7.12-7.09 (2H, m), 4.26 (2H, t, *J* = 6.6), 3.19-3.11 (1H, m), 2.59-2.51 (1H, m), 2.17-2.07 (2H, m), 1.90 (2H, dd, *J* = 13.6, 2.8), 1.80-1.73 (2H, m), 1.66 (2H, dd, *J* = 13.3, 3.2), 1.54-1.40 (4H, m), 1.36-1.16 (12H, m), 0.80 (3H, t, *J* = 6.7). ^13^C NMR (75 MHz, CDCl_3_) δ 185.5, 181.9, 158.1, 146.0, 138.6, 133.7, 133.0, 132.4, 131.5, 128.4, 128.2, 126.3, 125.9, 73.7, 43.4, 35.4, 34.5, 31.9, 30.4, 29.9, 29.7, 29.6, 29.4, 29.3, 26.0, 22.7, 14.1.

**Synthesis of ATO-C_4_**

*Butyl-ATO (*ATO-C_4_) was prepared by reacting bromobutane with ATO in the presence of potassium carbonate in DMF (Figure S1).

Bromobutane (0.15 g, 1.1 mmol) was added to a mixture of ATO (0.4 g, 1.1 mmol) and potassium carbonate (0.15 g, 1.1 mmol) in DMF (3 mL). The mixture was stirred at 70°C for 7 h. Diethyl ether was added to the mixture followed by water (20 mL). The organic layer was washed twice with water and dried over Na_2_SO_4_. The solvent was removed under reduced pressure and purification of the compound by flash chromatography (pentane/ethyl acetate, 98:2) yielded the product (0.38 g, 83%).

HRMS calculated for ATO-C_4_ C_26_H_27_ClO_3_ [MH]^+^ 423.1721, found, 423.1718.

^1^H NMR (400.13 MHz, CDCl_3_),δ 8.01-7.90 (2H, m), 7.63-7.55 (2H, m), 7.19-7.16 (2H, m), 7.11-7.08 (2H, m), 4.30 (2H, t, *J* = 6.7), 3.17-3.10 (1H, m), 2.57-2.49 (1H, m), 2.16-2.05 (2H, m), 1.90 (2H, dd, *J* = 13.5, 2.82), 1.79-1.71 (2H, m), 1.65 (2H, dd, *J* = 13.3, 3.2), 1.51-1.41 (4H, m), 0.94 (3H, t, *J* = 7.3). ^13^C NMR (75 MHz, CDCl_3_) δ 185.4, 181.8, 158.0, 145.9, 138.6, 133.7, 133.0, 132.4, 131.7, 128.4, 128.1, 126.3, 125.9, 73.7, 43.3, 35.4, 34.5, 32.4, 29.9, 29.9, 19.1, 13.8.

**Supplementary Figures**

**Figure S1. Synthetic schemes for Mito-ATO and alkyl ATO analogs.** Reagents and conditions: i, K_2_CO_3_, DMF, 70°C, 7-12 h.

**
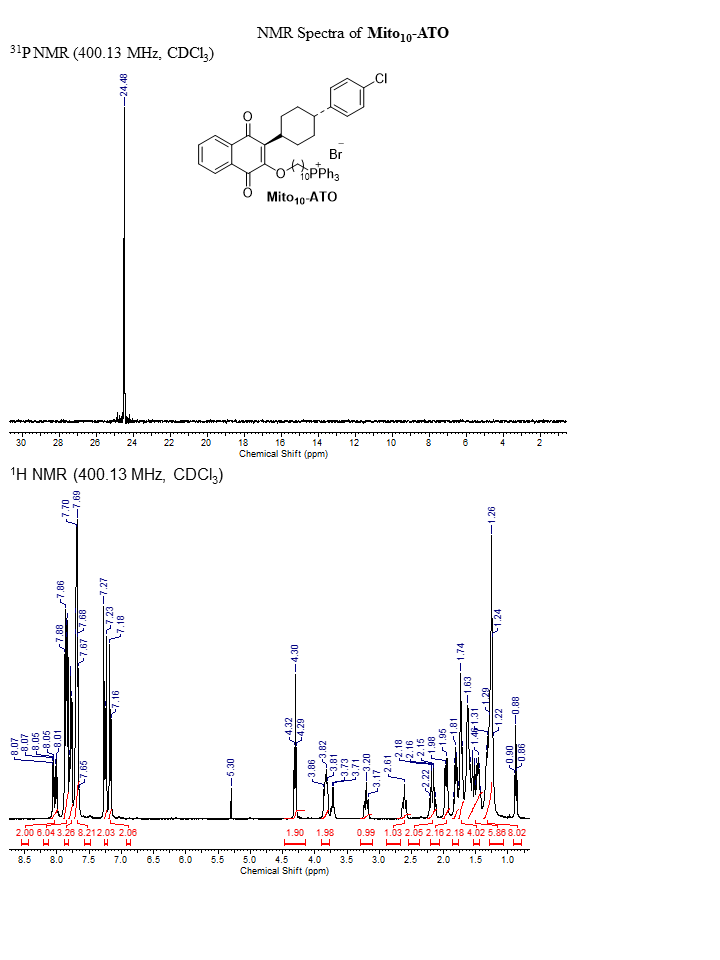
**

**
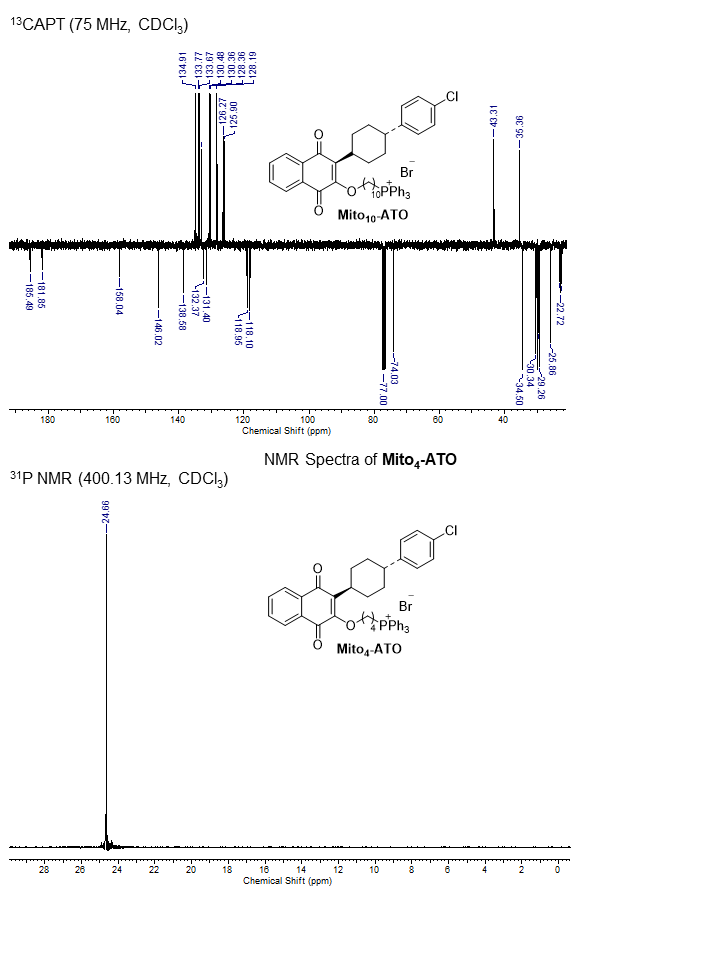
**

**
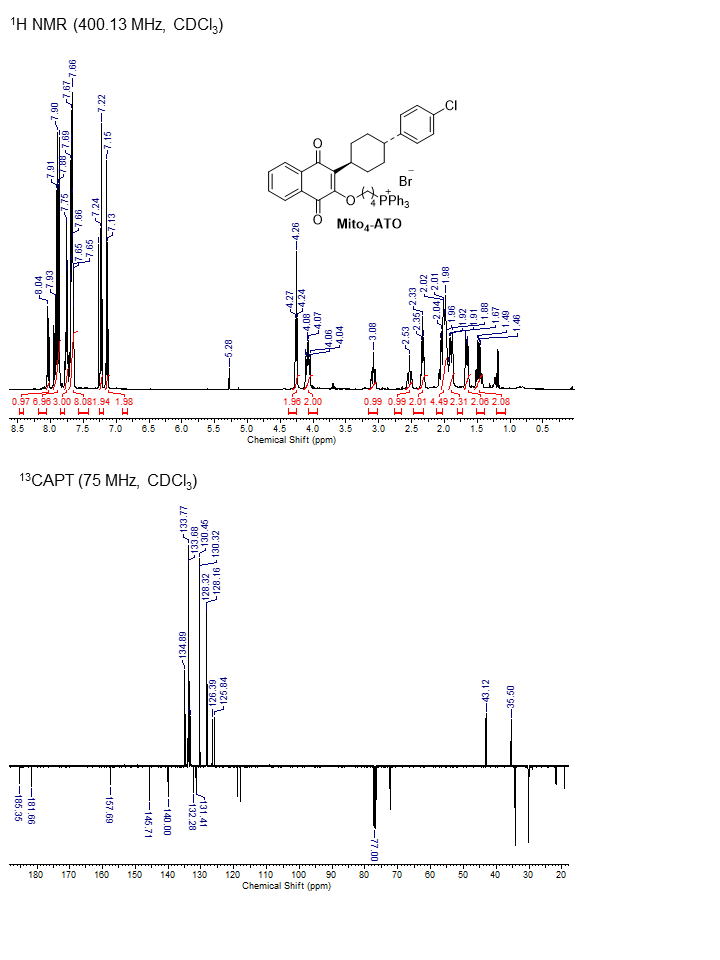
**

**
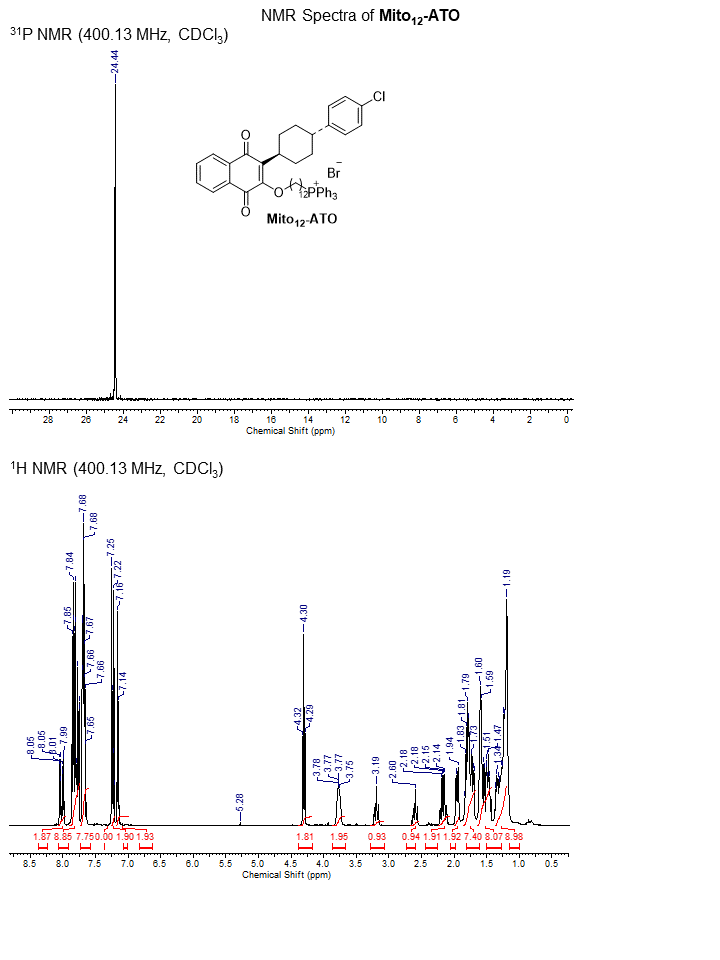
**

**
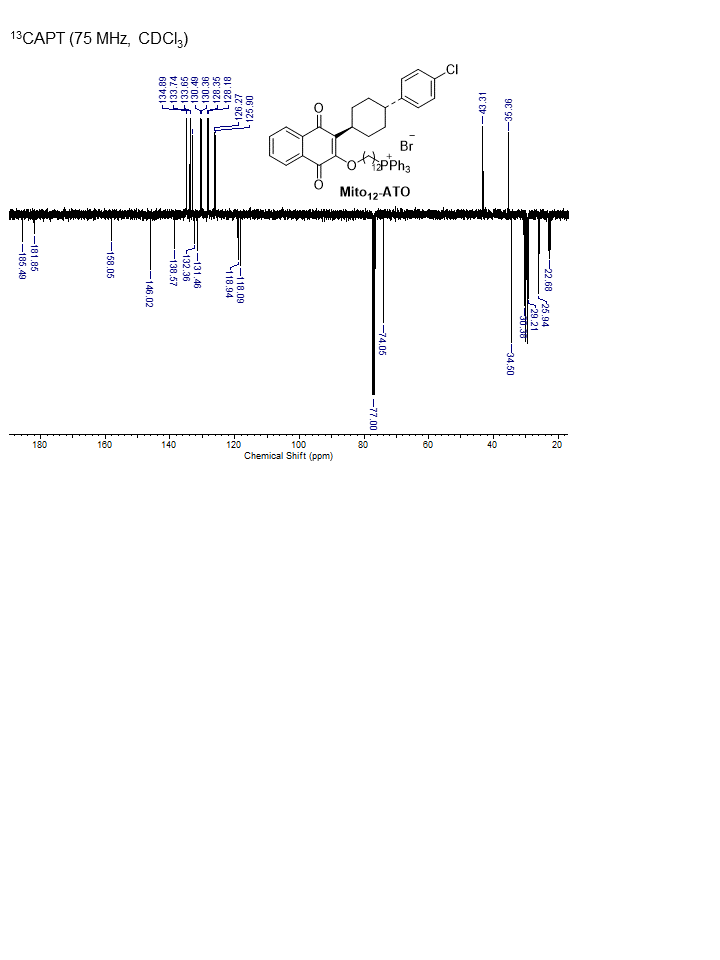
**

NMR Spectra of **Mito_16_-ATO**

^31^P NMR (400.13 MHz, CDCl_3_)

^1^H NMR (400.13 MHz, CDCl_3_)

^13^CAPT (75 MHz, CDCl_3_)

**
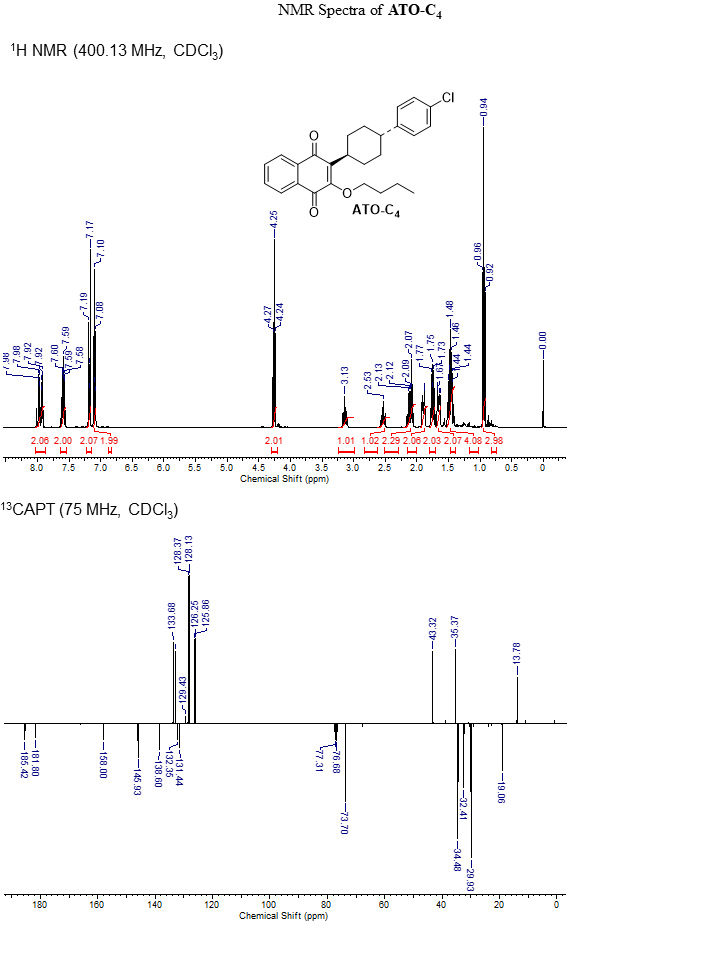
**

**
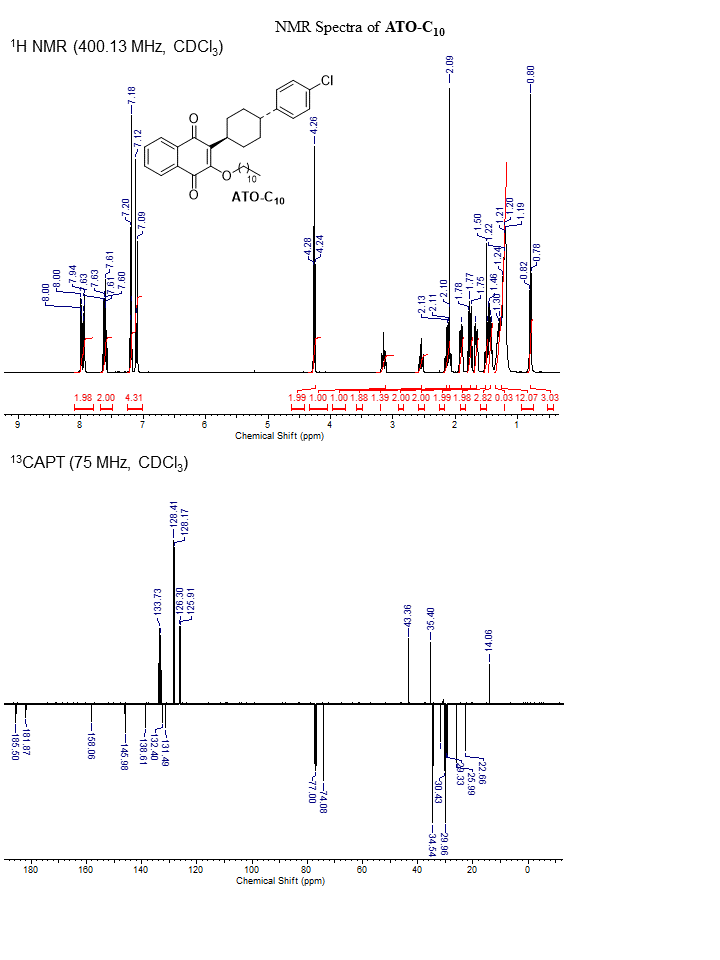
**

**Figure S2. NMR spectra and parameters of Mito-ATO analogs and alkyl-ATO analogs.**

**
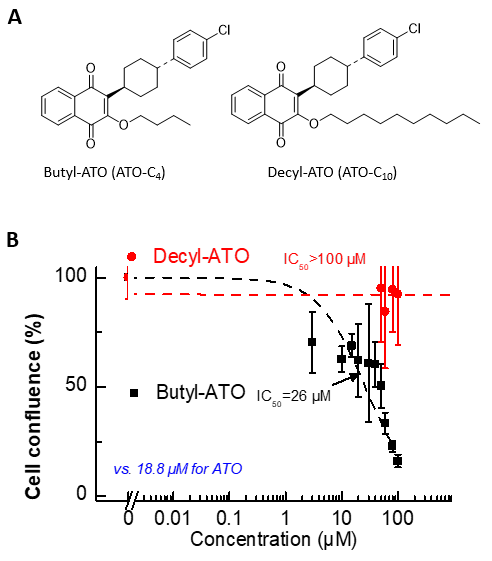
**

**Figure S3. Effects of ATO-C_4_ and ATO-C_10_ on proliferation of MiaPaCa-2 cells. (A)** Chemical structures of ATO containing a four-carbon alkyl chain and a 10-carbon alkyl chain. **(B)** Effects of ATO-C_4_ and ATO-C_10_ on the proliferation of MiaPaCa-2 cells were monitored as shown in Fig. 2. Cell confluence is plotted against concentration and the dashed lines represent the fitting curves used to determine the IC_50_ values.

**
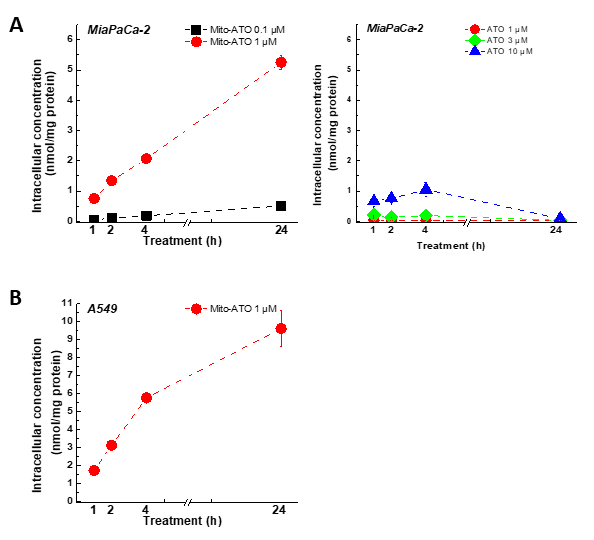
**

**Figure S4. Cellular uptake of Mito-ATO and ATO. (A)** Pancreatic cancer cells, MiaPaCa-2, were treated with Mito_10_-ATO **(left)** and ATO **(right)** for 1, 2, 4, and 24 h. Cellular uptake of Mito_10_-ATO and ATO was quantified using LC-MS/MS. **(B)** Cellular uptake of Mito-ATO in lung cancer cells, A549, was quantified using LC-MS/MS.
